# Immune-Endocrine Links to Gregariousness in Wild House Mice

**DOI:** 10.3389/fnbeh.2020.00010

**Published:** 2020-02-05

**Authors:** Patricia C. Lopes, Esther H. D. Carlitz, Morgan Kindel, Barbara König

**Affiliations:** ^1^Schmid College of Science and Technology, Chapman University, Orange, CA, United States; ^2^Department of Psychology, Biological Psychology, Technical University of Dresden, Dresden, Germany; ^3^Department of Evolutionary Biology and Environmental Studies, University of Zurich, Zurich, Switzerland

**Keywords:** sickness behaviors, cytokines, progesterone, corticosterone, testosterone, tumor necrosis factor, interferon, interleukin

## Abstract

Social interactions are critically important for survival and impact overall-health, but also impose costs on animals, such as exposure to contagious agents. The immune system can play a critical role in modulating social behavior when animals are sick, as has been demonstrated within the context of “sickness behaviors.” Can immune molecules affect or be affected by social interactions even when animals are not sick, therefore serving a role in mediating pathogen exposure? We tested whether markers of immune function in both the blood and the brain are associated with gregariousness, quantified as number of animals interacted with per day. To do this, we used remote tracking of social interactions of a wild population of house mice (*Mus musculus domesticus*) to categorize animals in terms of gregariousness. Blood, hair, brain and other tissue samples from animals with extreme gregariousness phenotypes were collected. We then tested whether the levels of three important cytokines (TNF-α, IFN-γ and IL-1β) in the serum, cortex and hypothalamus of these animals could be explained by the gregariousness phenotype and/or sex of the mice. Using the hair as a long-term quantification of steroid hormones, we also tested whether corticosterone, progesterone and testosterone differed by social phenotype. We found main effects of gregariousness and sex on the serum levels of TNF-α, but not on IFN-γ or IL-1β. Brain gene expression levels were not different between phenotypes. All hair steroids tended to be elevated in animals of high gregariousness phenotype, independent of sex. In sum, elements of the immune system may be associated with gregariousness, even outside of major disease events. These results extend our knowledge of the role that immune signals have in contributing to the regulation of social behaviors outside periods of illness.

## Introduction

One of the most powerful ways to alter the social behavior of animals is to make them sick. Infected animals behave in astonishingly different ways from healthy versions of themselves, displaying altered eating, drinking, sleeping, and sexual and social interaction patterns (Kent et al., 1992). We now know that the majority of these behavioral alterations is caused by the inflammatory response that takes place during an infection. Upon infection, receptors on the membranes of monocytes/macrophages recognize invaders, activating a signaling cascade that is associated with production and release of proinflammatory cytokines (Dantzer, 2017). This peripheral message reaches the brain in multiple ways and activates neural responses, including the local production of cytokines (Dantzer, 2017).

Given the extensive set of effects that proinflammatory cytokines can have on the behavior of sick animals, it is not surprising that links between inflammation and social dysfunction have been uncovered. For instance, autism spectrum disorder, schizophrenia and bipolar disorder, which are all disorders with some degree of social dysfunction, are associated with immune dysfunction involving altered balance of cytokine production (Monji et al., 2009; Altamura et al., 2014; Gottfried et al., 2015; Khandaker et al., 2015; Bhattacharya et al., 2016). Clinical depression, which frequently involves feelings of social disconnection, is more prevalent in people with illnesses accompanied by prolonged inflammation (Dantzer et al., 2008). Similarly, long-term activation of the immune system, such as during cancer, systemic infections or autoimmune disorders, can lead to the development of depressive symptoms in susceptible individuals. Studies in mice have shown that social interactions alter proinflammatory cytokine gene expression (interleukin-1, IL-1, and tumor necrosis factor-alpha, TNF) in the hypothalamus of endotoxin injected males (Weil et al., 2006). More recently, it was shown that mice deficient in interferon-gamma (*Ifng*^–/–^ mice) or its receptor (*Ifngr1*^–/–^ mice) present social deficits, characterized by absence of preference for a conspecific over an object (Filiano et al., 2016). Targeted deletion of the IFN-γ receptor in prefrontal cortex neurons was sufficient to alter social behavior, leading to a lack of social preference, highlighting involvement of IFN-γ signaling in the brain in the maintenance of normal social behavior.

Some authors have proposed that cytokines may be used beyond contexts of disease in helping to mediate social behaviors (Hennessy et al., 2014; Eisenberger et al., 2017; Lopes, 2017). The rationale is that, while social interactions are critical for survival and reproduction in many species, social aggregation increases exposure to socially transmitted pathogens. Therefore, immune signaling molecules may have been co-opted throughout evolution to impact and/or respond to social interactions. In our previous work with mice in a free-ranging population, we have found that an inflammatory challenge leads mice to drop social ties to their social group (Lopes et al., 2016). Here, we used a wild population of house mice (*Mus musculus domesticus*) to test for the presence of associations between central and peripheral cytokine production and the propensity to interact socially (gregariousness). We focused on two brain areas, the hypothalamus and the prefrontal cortex, that are important for social interactions (Ko, 2017) and where cytokine production has been shown to occur (Rothwell and Luheshi, 2000; Vitkovic et al., 2000; Monteiro et al., 2017). The cytokines studied here, TNF-α, IFN-γ and IL-1β, have been previously found to impact behavior (Dantzer, 2004; Filiano et al., 2016). In addition to this, as steroids impact cytokine production (Trigunaite et al., 2015; Dantzer, 2017) and behavior (Nelson and Kriegsfeld, 2017), we used a novel method to assess long-term hormone secretion of three steroids in hair and we tested whether those steroids correlated with social behavior and/or serum cytokine levels.

## Materials and Methods

Animal use and experimental design were approved by the Veterinary Office Zürich, Switzerland (Kantonales Veterinäramt Zürich, no. ZH091/16). Animal sampling was carried out in accordance with the Veterinary Office Zürich guidelines and is subject to the Swiss Animal Protection Law (TschG).

### Study Population

We studied a wild house mouse population located in a barn near Zurich, Switzerland. This population of free-living mice has been studied by Barbara König and colleagues since 2002 (for a detailed description see König and Lindholm, 2012). The barn consists of a 72 m^2^ building separated by plastic dividers into 4 sectors connected by holes and wooden sticks. The mice therefore have access to the entire space, but there is some structuring to the environment. Each sector contains four water bottles and three food trays, where water and food are provided *ad libitum*. Nest building materials (straw and hay) are also made available throughout the barn. The walls of the building contain small holes, allowing the mice to freely move in and out, but keeping larger predators (e.g., cats, foxes, or owls) out. This setup is designed to mimic the environment experienced by commensal mice in central Europe.

### Automated Collection of Social Behavior

Each sector of the barn contains 10 artificial nest boxes. Each nest box can be accessed by the mice via a cylindrical tunnel fitted with two round antennas (RFID readers). All mice in the population are captured every 6–8 weeks. During those events, mice are weighed, and several metrics are documented, including, sex, reproductive status, ectoparasite load and presence of wounds. Finally, mice at or over 18 g are implanted subcutaneously with a unique RFID tag (Trovan ID-100, Euro ID Identifikationssysteme GmbH & Co, Germany), and an ear punch is taken for genetic analysis. Every time a tagged mouse goes in or out of a nest box, its identity is automatically recorded by the antennas, along with a timestamp, and the information is transferred to a laptop located inside the barn. At the end of each day, the information on all movements for each logged mouse relative to the 40 nest boxes is automatically transferred to a centralized database. This automated recording system is described in detail in König et al., 2015 and allows us to calculate, per logged individual, the number of nest boxes used, time spent in each nest box, and the identities of each animal that overlapped in time in any given box (Lopes et al., 2016).

The sampling for this study took place during late December to February, falling in the non-reproductive season. We used this period to minimize the number of untagged individuals (as pups and juveniles under 18 g are not tagged) and to avoid confounding effects of reproductive activity. We constructed social co-location networks for each 24 h of data for time overlap in nest box use. To identify the components (social groups) existing in the barn and visualize the social co-location networks we used UCINET (Borgatti et al., 2002) and Netdraw (Borgatti, 2002), respectively. For each 24 h period of time, we calculated a degree value for each logged animal, which consisted of the number of total individuals encountered in nest boxes within that day. We used five 24 h periods of time to calculate the distribution of degrees for the population, and the mean degree ± 1 STD. To define the high and low social gregariousness phenotypes in the population to be sampled, we focused on mice that were consistently (over 3 separate days) at least either 1 STD above (high gregariousness) or below (low gregariousness) the mean degree of the population. These calculations were done prior to each sampling day and the IDs of mice to be sampled along with the nest boxes they regularly visited would be highlighted for the target day. Using these criteria, we were able to capture and sample 7 females of each social gregariousness phenotype. For males, it was not possible to find enough individuals that were above 1 STD of the mean, so we targeted the males that most approached that value. In total, we sampled 8 low gregariousness males and 7 high gregariousness males. Mouse capture and tissue sampling procedure is described in section “Tissue Sampling and Carcass Analysis.” For the purposes of statistical analysis, the gregariousness degree value for each mouse is the average degree over three separate days.

### Tissue Sampling and Carcass Analysis

Each sampling day, we arrived at the barn within 2 h after sunrise. We immediately plugged the entrance tubes to the nest boxes used by the target animals. Using a handheld RFID reader, we identified which nest boxes the target animals were in and unplugged the other ones. We then used a glass jar to remove each mouse out of the box sequentially, until the focal mouse was present in the jar and identified using the handheld RFID reader. This mouse was then brought in the jar to a processing station on site, where it was weighed and then euthanized via CO_2_ inhalation. The time between entering the focal mouse sector and euthanasia was recorded for each mouse. The mice were then decapitated using scissors, trunk blood was collected into a 2-mL Eppendorf tube, and the brain was removed from the skull onto a piece of aluminum foil and immediately placed on dry ice. Afterward, the liver, spleen and uterus/testis were dissected and also preserved on dry ice on a piece of foil. The carcasses were then inspected for the presence of wounds and visible ectoparasites. Finally, an ear punch sample was collected and preserved in ethanol and a sample of hair from the lower back was shaved off and preserved in a paper envelope. At arrival at the University of Zurich, the blood samples were spun down for 10 min and the serum portion was preserved in a separate tube at −80°C.

### Serum Cytokine and Corticosterone Quantification

To quantify levels of important circulating cytokines in the serum, we used the Mouse Cytokine Magnetic Panel (MCYTOMAG-70K-03) MILLIPLEX^®^ MAP kit for three target analytes: IFN-γ, IL-1β, and TNF-α. The samples were run in duplicate at a volume of 12.5 μL of serum per well diluted 1:2 in Assay Buffer, following manufacturer’s instructions. The wells were read using a Magpix^®^ system. The detection limits for each analyte were (in pg/mL): 3.05 for IFN-γ, 0.81 for IL-1β and 4.02 for TNF-α. The intra-assay % CV for replicates for each analyte was 11% for IFN-γ, 8% for IL-1β, and 9% for TNF-α.

Serum corticosterone was quantified using the Arbor Assays DetectX^®^ ELISA kit (K014-H1). Samples were diluted 1:100 and run in duplicate, following manufacturer’s instructions (including use of the Dissociation Reagent). The wells were read using a Tecan Spark microplate reader. The detection limit was 16.9 pg/mL and the intra-assay % CV for replicates was 6%.

### Hair Sample Preparation and Hormone Analysis

Hair samples were shaved from the lower back of the animals with an electric razor after euthanasia (∼15 – 20 mg hair, equivalent to a square of 2 cm × 1 cm). Samples were kept in individual paper envelopes at room temperature until analysis. Hair sample preparation followed the protocol as described for human hair (Gao et al., 2013) with slight adaptations, which were necessary to prevent loss of very short and fine mouse hair (∼5 mm) during washing. Hair was washed once by shaking it in 1.5 mL of isopropanol for 3 min at room temperature after which the liquid was pipetted off. Hair was then allowed to dry for at least 12 h at room temperature. 5 ± 0.5 mg of non-pulverized hair was transferred into a 3-mL glass tube. Thereafter, 1.8 mL methanol was added, and the hair was incubated for 18 h at room temperature for steroid extraction. 1.6 mL of the clear supernatant was transferred into a new 2-mL plastic tube (Eppendorf, Hamburg, Germany). The alcohol was evaporated at 50°C under a constant stream of nitrogen until the samples were completely dried (duration: approximately 40 min). The dry residue was suspended using 225 μL distilled water (LC–MS-grade) and 25 μL methanol. 100 μL of the re-suspension, together with 20 μL of an internal standard were used for liquid chromatography–tandem mass spectrometry (LC–MS/MS) analysis. The LC–MS/MS analysis followed a validated protocol for corticosterone, testosterone and progesterone (Gao et al., 2013). The biological validity of these hair steroids was recently assessed for mice from the same population (Carlitz et al., 2019).

### Brain Cytokines: Gene Expression Quantification

#### Dissection of Brain Regions

The three brain regions of interest collected as part of a larger project were the prefrontal cortex, the hypothalamus and the hippocampus, and the regions were identified through use of the Allen Mouse Brain Atlas: P56, Coronal Reference Atlas (Lein et al., 2007). Frozen brains were sectioned coronally on a Leica CM1860UV cryostat at −18°C and the brain regions of interest were extracted using surgical micropunches (EMS Rapid Core Instruments). First, we used Tissue-Plus O.C.T. Compound (Fisher, catalog # 23-730-571) to attach the frozen brain to a specimen disc, positioned coronally relative to the blade, as similar as possible to the positioning described in the Atlas. Note regarding the prefrontal cortex: because there is debate regarding the boundaries of this region in mice (Carlén, 2017), we opted to collect the more rostral portion of the entire cortex. To do this, we punched out and discarded any region not marked as Cortex in the Allen Atlas (within the coordinates indicated below) using a 7 mm punch and collected what was left. We collected samples from the prefrontal cortex region (approximate coordinates: bregma 3.245 to 0.845 mm), from the hypothalamus (approximate coordinates: bregma 0.845 to −3.08 mm; punch size: 3 mm) and from the hippocampus (approximate coordinates: bregma −1.055 to −5.055 mm; punch size: 3 mm). Punches for the specific regions were obtained from 100 μm slices, spaced out by two 20 μm slices (one of which was collected for histology on microscope slides Fisherbrand, item 12-550-15). Punches were preserved in 1 mL of Trizol reagent (Ambion, item 149204) in three separate 2-mL tubes (one per region) containing zirconium 1.5 mm size beads (Benchmark Scientific, item D1032-15). Once all punches were collected, the tissue was homogenized by placing the tubes in a bead-mill homogenizer (Beadbug 6 homogeneizer, Benchmark Scientific) for 20 s at an agitation speed of 4 ms^–1^. After 5 min of rest time, the liquid was transferred to a new tube and frozen at −80^°^C until further use.

For the present study, we used only the prefrontal cortex and the hypothalamus samples.

#### Quantitative RT-PCR

Total RNA was obtained from the chloroform extracted aqueous phase using the Zymo Direct-zol RNA Miniprep Plus kit (Zymo, catalog # R2071) following manufacturer’s instructions, including the DNase I in-column treatment. RNA quantity and purity were assessed on a Nanodrop instrument. Reverse transcription was done on 500 ng of total RNA using the iScript RT Supermix kit (Bio-Rad, catalog # 1708841). Quantitative real-time PCR was done on a CFX instrument (Bio-Rad), using SSO Advanced Universal SYBR (Bio-Rad, catalog # 1725275) and the thermocycling parameters according to manufacturer’s instructions (annealing temperature of 60^°^C). PCR reactions were run in a total volume of 10 μL, containing 3 μL of 1:10 diluted cDNA, 0.25 μL (except IL-1β, which was 0.5 μL) of each 10 μM primer, 5 μL of SYBR and water up to 10 μL. The following gene specific primers were used: IFN-γ (F: 5′-ATGAACGCTACACACTGCATC-3′; R: 5′-CCATCCTTTTGC CAGTTCCTC-3′), IL-1β (F: 5′-TGCCACCTTTTGACAGT GATG-3′; R: 5′-ATGTGCTGCTGCGAGATTTG-3′), TNF-α (F: 5′-GCTGAGGTCAATCTGCCCAA-3′; R: 5′-GGGGCTCT GAGGAGTAGACA-3′), and β-Actin (F: 5′-GGCTGTATTCC CCTCCATCG-3′; R: 5′-CCAGTTGGTAACAATGCCATGT-3′). Primer specificity was tested by sequencing of PCR products (GenScript United States Inc). Gene expression raw fluorescence readings were first submitted to PCR Miner online software (Zhao and Fernald, 2005) for computation of mean cycle thresholds (*Ct*) and gene amplification efficiency (*E*). Using these values, we calculated the *R*_0_ (the start template concentration) for each sample for each gene according to the formula (Zhao and Fernald, 2005):

$$R_{o}=\frac{1}{{\left(1+E\right)}^{\mathrm{Ct}}}$$

For normalization, the *R*_0_ for genes of interest for each sample were divided by the corresponding *R*_0_ for the housekeeping gene β-Actin. The normalized *R*_0_ values were used for the statistical analyses.

### Statistical Analysis

All analyses were done in R version 3.5.2 (R Core Team, 2017). Graphs were prepared using base R function or the ggplot2 package (Wickham, 2016). As the data deviated from the assumptions required to run parametric tests (including residuals with non-normal distributions and potential outliers), we used non-parametric tests to assess the effects of Sex, Social Gregariousness Phenotype and their interaction on the variables of interest. This was done by running robust two-way rank-based ANOVAs by use of the function raov, contained in the Rfit package (Kloke and McKean, 2012). A correlation between serum corticosterone and time taken to capture the mice was tested using Spearman rank correlations. For boxplot graphs: each box represents the interquartile range, with the middle line marking the median; the whiskers represent the largest value within 1.5 times above the 75th percentile (top whisker) and below the 25th percentile (bottom whisker); the points represent outliers (any value 1.5 times higher or 3 times lower than the interquartile range beyond either end of the box).

## Results

### Social Behavior

**FIGURE 1:**
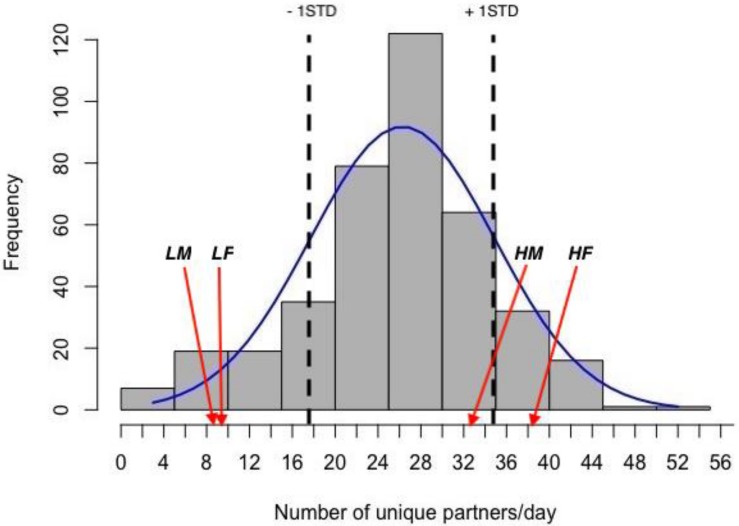
Frequency distribution of daily degree values (number of partners met in nest boxes each day) in the population and normal distribution curve. The values for 1STD of the mean are marked with dotted vertical lines. The positions of the mean values for high (*H*) and low (*L*) gregariousness male (*M*) and female (*F*) groups used in this study are indicated by arrows.

The mean degree of gregariousness for the population during the study period was (mean ± STD) 26 ± 8.6 partners/day (Figure 1). Mean degree values (mean ± SEM) and mean ages in days (±SEM) for sampled animals are given in Table 1. For sampled animals, there was no significant difference in age between the sexes (*F* = 1.41, *p* = 0.245), nor between gregariousness phenotypes (*F* = 1.44, *p* = 0.24), or their interaction (*F* = 2.37, *p* = 0.136).

**TABLE 1:** Summary statistics for degree (number of daily social partners) and age (in days) for captured animals of both sexes belonging to two gregariousness phenotypes (high or low gregariousness).

Gregariousness phenotype	Sex	Mean ± SEM	
		Degree	Age
High	Males	32.5 ± 1.3	246.6 ± 68.1
	Females	38.1 ± 2.5	207.2 ± 57.7
Low	Males	8.7 ± 2	125.8 ± 45.6
	Females	9.5 ± 1.3	250.4 ± 63.2

### Blood Levels of Cytokines and Corticosterone

**FIGURE 2:**
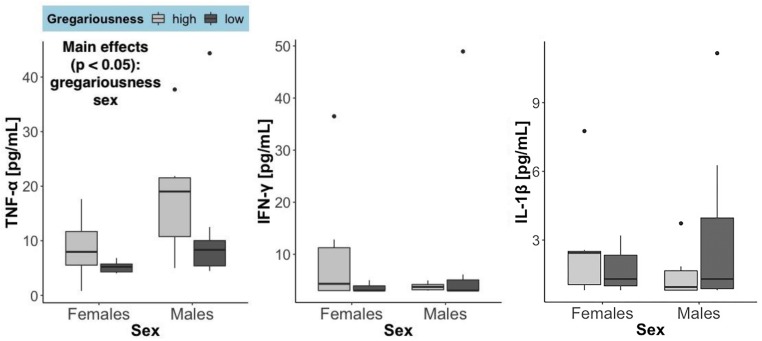
Serum levels of the three cytokines quantified, summarized by sex and gregariousness phenotype. High gregariousness data are represented by light gray boxes and low gregariousness by dark gray boxes. Please refer to section “Materials and Methods” for details on what the box plots represent. Sample sizes are 7 females of each gregariousness phenotype (14 total) and 8 males of low and 6 males of high gregariousness phenotype (14 total).

There was a significant effect of sex (*F* = 12.5, *p* = 0.00161) and of gregariousness phenotype (*F* = 10.89, *p* = 0.00291) on circulating levels of TNF-α, as well as a significant interaction between these two terms (*F* = 5.038, *p* = 0.0339). No significant main effects or interactions were found for any of the other cytokines quantified (*p* > 0.05). For all cytokines, one high gregariousness male had extremely high values that were 17.5 (TNF-α: 201.71 pg/mL), 16.5 (IFN-γ: 260.58 pg/mL) and 14.3 (IL-1β: 63.47 pg/mL) times higher than the overall mean for each cytokine. Removal of this outlier did not change the conclusions for IFN-γ nor IL-1β, but altered the ones for TNF-α. While TNF-α levels were still found to be higher in males (main effect of sex: *F* = 7.2, *p* = 0.0013) and higher in animals with high gregariousness phenotype (main effect of phenotype: *F* = 8.73, *p* = 0.0069), the interaction term was now deemed non-significant (*F* = 2.92, *p* = 0.1). In light of this, we here present and interpret the cytokine results without the outlier (Figure 2).

**FIGURE 3:**
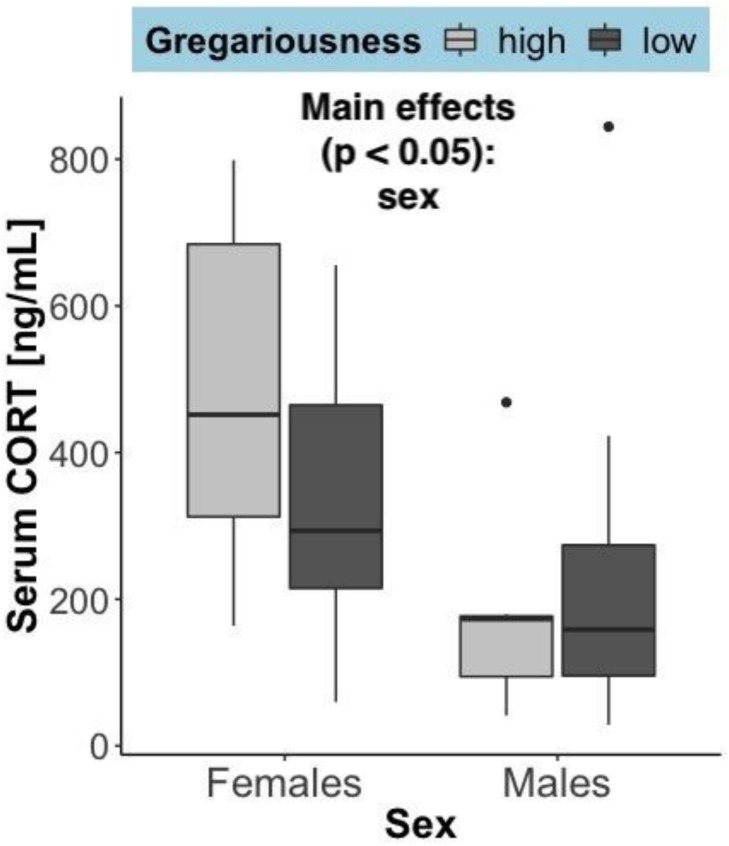
Serum corticosterone (ng/mL) at time of capture, summarized by sex and gregariousness phenotype. High gregariousness data is represented by light gray boxes and low gregariousness by dark gray boxes. Please refer to section “Materials and Methods” for details on what the box plots represent. Sample sizes are 7 females of each gregariousness phenotype (14 total) and 8 males of low and 7 males of high gregariousness phenotype (15 total).

Time to euthanasia (mean ± SEM) was not significantly different between sexes (females = 9.71 min ± 2.81 and males = 9.64 min ± 2.03; *F* = 0.021, *p* = 0.885), gregariousness phenotypes (high = 10.86 min ± 3.06 and low = 8.5 min ± 1.55; *F* = 0.021, *p* = 0.885), or their interaction (*F* = 2.23, *p* = 0.148). The time it takes from entering the barn to euthanasia can influence circulating levels of corticosterone. As expected, there was a significant positive correlation between serum corticosterone and time until euthanasia: *r*_*s*_(27) = 0.57, *p* = 0.00172. Serum corticosterone (Figure 3) was significantly higher in females (*F* = 7.14, *p* = 0.0133), but did not differ due to gregariousness phenotype (*F* = 0.852, *p* = 0.365) nor due to a sex by gregariousness interaction (*F* = 1.62, *p* = 0.214). Serum corticosterone was not significantly correlated to serum levels of any of the cytokines (*p* > 0.05 for all correlations).

### Long-Term Steroid Hormone Levels

**FIGURE 4:**
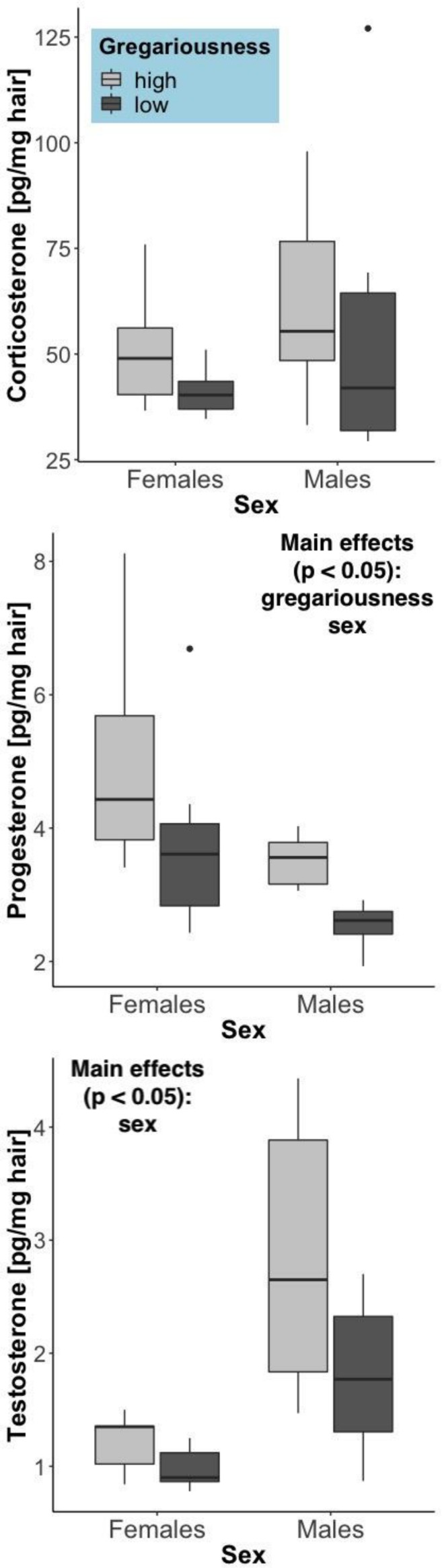
Steroid hormone concentrations in hair (pg/mg hair), summarized by sex and gregariousness phenotype. High gregariousness data is represented by light gray boxes and low gregariousness by dark gray boxes. Please refer to section “Materials and Methods” for details on what the box plots represent. Sample sizes are 7 females of each gregariousness phenotype (14 total) and 8 males of low and 7 males of high gregariousness phenotype (15 total).

The hair steroid measurements reflect a long-term accumulation (over months; Carlitz et al., 2019) of these hormones. Differently from the serum corticosterone levels (which reflect secretion over the last several minutes), hair corticosterone showed no significant difference between the sexes (*F* = 0.908, *p* = 0.345), a non-significant trend toward elevation in high gregariousness phenotypes (*F* = 3.40, *p* = 0.077) and no significant interaction of these terms (*F* = 0.466, *p* = 0.50). As expected, significant sex differences were found for the other two steroid hormones (Figure 4). Testosterone was higher in males (*F* = 15.03, *p* = 0.00072) and progesterone was higher in females (*F* = 10.42, *p* = 0.0035). An effect of gregariousness was present for progesterone (*F* = 11.62, *p* = 0.0022) and a trend for testosterone (*F* = 4.13, *p* = 0.053), with both steroids being elevated in the high gregariousness phenotypes. No significant interactions between sex and gregariousness phenotypes were found for either testosterone (*F* = 1.49, *p* = 0.234) or progesterone (*F* = 0.0122, *p* = 0.913).

### Brain Cytokine Expression

No significant effects of sex, gregariousness phenotype or their interaction were found for the cytokines quantified in the hypothalamus or in the prefrontal cortex (Table 2).

**TABLE 2:** Robust ANOVA test results for gene expression levels for the housekeeping gene (β-Actin, raw *R*_0_ values used for statistical test) and for the genes of interest (normalized *R*_0_ values used; see section “Materials and Methods” for *R*_0_ and normalization calculation details) in the two brain regions studied.

		Hypothalamus			Cortex		
Response	Variables	Robust F	DF	*p*-value	Robust F	DF	*p*-value
β-Actin	Sex	0.00646	1	0.93659	0.04644	1	0.83112
	Gregariousness	0.16458	1	0.68857	2.81706	1	0.10573
	Sex^∗^Gregariousness	0.01381	1	0.90743	2.63477	1	0.11709
TNF-α	Gregariousness	0.10403	1	0.74996	1.24576	1	0.27498
	Social	0.43879	1	0.51429	1.25199	1	0.27381
	Sex^∗^Gregariousness	0.46462	1	0.50228	0.688	1	0.4147
IFN-γ	Gregariousness	3.46167	1	0.0751	2.84643	1	0.10402
	Social	0.17499	1	0.67943	0.0099	1	0.92155
	Sex^∗^Gregariousness	0.122	1	0.72992	0.81287	1	0.37588
IL-1β	Gregariousness	2.02625	1	0.16747	1.3115	1	0.26297
	Social	0.11621	1	0.73615	0.73205	1	0.40034
	Sex^∗^Gregariousness	0.08409	1	0.77432	2.29556	1	0.14229

*Sample sizes are 7 females of each gregariousness phenotype (14 total) and 8 males of low and 7 males of high gregariousness phenotype (15 total).*

## Discussion

In this study, we asked whether house mice on the extreme ends of a gregariousness distribution in a wild population showed differences in proinflammatory and steroid production profiles. We studied mice during the non-reproductive season in order to reduce effects related to reproductive behavior. We found that circulating TNF-α levels were higher in individuals with a high gregariousness phenotype, regardless of sex. Contrastingly, no differences in cytokine brain gene expression amongst social phenotypes were found. In terms of its potential to alter brain functioning and/or impact behavior, changes in the expression of TNF-α in the brain are not necessary as this cytokine is known to be able to cross the blood brain barrier (Banks et al., 1995). The direction of these results is surprising because TNF-α elevations (in the context of immune challenges or disease) are associated with the induction of sickness behaviors, which include decreased social interactions (Dantzer, 2004). It is important to note, however, that the serum TNF-α levels reported here are several orders of magnitude lower than the levels observed in the acute phase of an immune challenge. For example: a mean of 750 pg/mL TNF-α is found in plasma 2 h post-endotoxin injection in male mice (Copeland et al., 2005) while the highest observation in Figure 2 of our study is an outlier with a value less than 40 pg/mL. Relatively high baseline levels of TNF-α may thus exert different effects than those elicited by disease-induced levels of this cytokine. Current evidence points toward important roles of TNF-α in central nervous system functioning and development (Bilbo and Schwarz, 2012), so TNF-α could be associated with gregariousness in different ways, such as through effects on learning and memory. Several relationships between social interactions and immune responses in humans (Uchino et al., 1996) and in non-human primates and rodents have been found (Takahashi et al., 2018). Specific to TNF-α, breast cancer patients reporting increased social satisfaction or social activities had stronger TNF-α responses to an endotoxin challenge (Marucha et al., 2005). In mice, exposure to chronic defeat can lead to alterations to the profile of immune cells present in the spleen, as well as changes in gene expression of those cells, resulting in increased production of TNF-α (Ambrée et al., 2018). Therefore, the immune system can respond to social interactions and affect changes in inflammatory cytokines. It is thus possible that, rather than being a driver of gregariousness, TNF-α was increased in mice with a high gregariousness phenotype as a result of social interactions. Such a relationship between gregariousness and TNF-α could be evolutionarily selected for in case TNF-α confers some adaptive value in this context, as the production of immune responses is costly in terms of energy and resources (Sheldon and Verhulst, 1996; Zuk and Stoehr, 2002; Viney et al., 2005). In humans, anti-TNF-α drugs lead to increased risk of contracting infectious diseases, such as tuberculosis (Wallis, 2009). Increased levels of TNF-α may therefore confer increased protection against infectious diseases (particularly those that require close individual contact).

When we used hair samples to quantify long-term secretion of steroid hormones, sex differences were found, with progesterone being higher in females and testosterone higher in males, as one would expect from basic mammalian physiology. This is an indication that using hair to assess cumulative steroid levels in mice is an appropriate method (see also Carlitz et al., 2019). Circulating (serum) corticosterone levels were higher in females than in males, even though there were no differences in capture time between males and females. This could be due to female mice having naturally higher baseline corticosterone than males (e.g., Malisch et al., 2007), which we cannot test given that not all of our serum samples were taken quickly enough (limit is 2–3 min). Another possibility is that females are more sensitive to human presence or to the stress of being captured, which would fit with findings in other species of a sex difference in glucocorticoid production in response to stressors (reviewed in Bangasser and Wiersielis, 2018).

More interestingly, for all steroid hormones quantified in hair, there was a tendency for elevated levels to be present in mice with a high gregariousness phenotype, with the strongest effect observed for progesterone. Two ways in which steroids and social behavior can be related are through steroid effects on behavior or, conversely, behavioral induction of steroid secretion. We will discuss both of these possibilities in the following paragraphs.

Research in humans has found progesterone to be associated with social closeness and affiliative motivation (Schultheiss et al., 2003; Brown et al., 2009). For instance, a task designed to increase closeness in a female dyad led to increased salivary progesterone in those subjects, whereas no increase in progesterone was found in participants after a neutral dyadic task (Brown et al., 2009). In female rodents, progesterone metabolites contributed to socio-sexual behaviors, including affiliation (Frye et al., 2006). In male rats, progesterone administration led to reduced social recognition memory, quantified as no difference in time investigating a novel or familiar conspecific (Bychowski and Auger, 2012). In this paradigm, social recognition was recovered by concomitant administration of a progestin receptor (PR) antagonist, indicating that progesterone exerts this effect through action on PRs. The ability of this steroid to change social recognition leading to lack of preferences between familiar and unfamiliar conspecifics could help explain its association with gregariousness in the current study. Besides promoting social closeness and reduced social recognition memory, high progesterone levels are associated with reduced aggression in male mice (Erpino and Chappelle, 1971) and non-maternal aggression in females of at least two species of rodents (Syrian hamsters and California mice; Kohlert and Meisel, 2001; Davis and Marler, 2003). Hormonal correlates of non-maternal aggression in female house mice have not been extensively studied, perhaps because laboratory strains of female mice are not prone to extensive aggression. We do however, find wild female house mice to both show aggressive behaviors and to be recipients of aggression, both in semi-natural enclosures (Carlitz et al., 2019) and in the lab (Harrison et al., 2016, 2017; Lopes et al., 2018; in all of these laboratory studies, several trials had to be interrupted due to excessive aggression). It is therefore possible that due to its effects in reducing aggression, high levels of progesterone facilitate life in larger social groups in house mice.

Animals that live in large social groups may also be more proficient at coping with social stress. The trend toward higher levels of corticosterone in higher gregariousness animals is an indicator that living at higher densities may be somewhat stressful. In experiments with male (Brockhurst et al., 2015) and female (Lyons et al., 2018) mice exposed to repeated same-sex resident strangers, the intruder mice exhibit increases in corticosterone and improved behavioral indicators of stress coping abilities.

It is difficult to determine whether the similar patterns observed for serum TNF-α and amount of steroid in hair (higher levels of all were found in high gregarious animals of both sexes) are connected given the different timelines reflected by those tissues. As proinflammatory cytokine production is affected by biological sex (Klein and Flanagan, 2016; Ter Horst et al., 2016) and by the predominant steroids for each sex (Bouman et al., 2005), a link is possible. In one study, testosterone was found to stimulate TNF-α production in both male and female rodents (Metcalfe et al., 2008). Progesterone has more consistently been found to have an immunosuppressive effect (Tait et al., 2008; Kadel and Kovats, 2018; Benagiano et al., 2019), which makes it a less evident underlying cause for the elevated TNF-α levels in individuals with high gregariousness phenotypes. Glucocorticoids (including corticosterone) can have both immunosuppressive and immunoenhancing effects, depending on factors such as levels, timing and duration (Sorrells and Sapolsky, 2007; Sorrells et al., 2009). For instance, acute exposure to glucocorticoids stimulates immune function, while chronic exposure is usually suppressive (Sorrells et al., 2009). In mouse models of social disruption, involving repeated aggressive interactions, elevated levels of proinflammatory cytokines are observed (including TNF-α; e.g., Avitsur et al., 2005; Bailey et al., 2009) even while serum corticosterone remains high (reviewed in Takahashi et al., 2018).

## Conclusion

Wild house mice show variation in their propensity to interact socially. In this descriptive study, we find that individuals with a high gregariousness phenotype also show high levels of the serum cytokine TNF-α and of progesterone quantified in the hair. A stronger immune response in animals engaging in a large number of social interactions could be evolutionarily advantageous by helping to prevent and/or recover from injuries and communicable diseases. We propose that high progesterone levels facilitate living in large social groups and that high baseline serum levels of TNF-α may occur as a consequence of social interactions and confer some protection from communicable diseases that accompanies living in close proximity to others. The causality of these ideas remains to be tested and offers ground for future studies.

## Data Availability Statement

The datasets generated for this study can be found in the Figshare repository, https://figshare.com/s/d7ce371e673768ca462b.

## Ethics Statement

The animal study was reviewed and approved by the Veterinary Office Zürich, Switzerland (Kantonales Veterinäramt Zürich, no. ZH091/16). Animal sampling was carried out in accordance with the Veterinary Office Zürich guidelines and is subject to the Swiss Animal Protection Law (TschG).

## Author Contributions

PL designed the experiment, conducted the research, collected the samples, and analyzed the data. EC developed the method and quantified the steroids in hair samples. MK helped to process the molecular data as well as the blood hormonal samples. BK started and maintained the study population, and contributed to the study design. All authors contributed toward preparing the manuscript.

## Conflict of Interest

The authors declare that the research was conducted in the absence of any commercial or financial relationships that could be construed as a potential conflict of interest.
